# Supplementary material for: Spinning-enabled wireless amphibious origami millirobot
Source: Nat Commun. 2022 Jun 14;13:3118. doi: 10.1038/s41467-022-30802-w (PMC9198078; doi:10.1038/s41467-022-30802-w)
Supplement: Supplementary file 1 — Supplementary Information [file 41467_2022_30802_MOESM1_ESM.pdf]

Supplementary Information for

Spinning-enabled Wireless Amphibious Origami Millirobot

Qiji Ze, Shuai Wu, Jize Dai, Sophie Leanza, Gentaro Ikeda, Phillip C. Yang, Gianluca Iaccarino, Ruike Renee Zhao

Ruike Renee Zhao
E-mail: rrzhao@stanford.edu

This PDF file includes:

Supplementary text
Supplementary Figs. 1 to 20
Supplementary Table 1
Legends for Supplementary Movies 1 to 12
SI References

Other supplementary materials for this manuscript include the following:

Supplementary Movies 1 to 12

1.Sample Fabrication

Kresling Origami. Geometries of four Kresling origami designs with different height-to-diameter aspect ratios (H/D) are provided in Supplementary Table 1. Kresling samples are folded from two-dimensional patterns using 0.05 mm thick polypropylene films, cut by a mechanical cutter (Cricut Maker, Cricut, Inc., USA). As shown in Supplementary Fig. 1a, the Kresling pattern (Design 2, $H = 4.82$ mm) with hole and cuts is folded from a two-dimensional flower-shaped pattern. After the pattern is folded, Mylar (0.127 mm thick) hexagons with and without a 3 mm hole are attached to the pattern's top and bottom sides, respectively, producing the Kresling with one frontal hole and six radial cuts. To illustrate the effects of the hole and cuts on promoting the swimming speed (Fig. 3d), the Kresling pattern (Design 2, $H = 4.82$ mm) without hole and cut is folded from a strip-shaped pattern, as shown in Supplementary Fig. 1b. Two Mylar hexagons without a hole are attached to the pattern's top and bottom sides.

In this work, Design 2 ($H = 4.82$ mm) is selected for all demonstrations in the main text based on the characterizations of mechanical properties, swimming performance, and magnetic folding of four Kresling designs, which will be analyzed in detail in the section "Selection of the Kresling Design for Demonstrations".

Surface Treatment of the Polypropylene Film for Water Penetration. Due to the small sizes of the Kresling robot's hole and cuts, water is not guaranteed to thoroughly fill the internal cavity of the robot when the working environment changes from ground to water, leading to uncontrollable robot density and swimming performance. To address this issue, we distinguish the outer and inner surfaces of the robot to be hydrophobic and hydrophilic for easy water penetration into the interior of the robot through the frontal and radial cuts based on the Janus membrane mechanism(1). Both sides of the untreated polypropylene film are initially hydrophobic. We first use sandpaper (180 Grit) to sand one side of the film uniformly. Then, we apply a layer of hydrophilic coating mixture (Hydrophilic Coating 8-3C, Coatings2Go LLC, USA) on the surface of this side. After curing at room temperature for 4h, the treated hydrophilic/hydrophobic polypropylene film is ready to be cut for Kresling fabrication.

Magnetic Plates. Two magnetic plate designs with different thicknesses are adopted for the magnetic actuation of the robots. The cavities are introduced in the magnetic plate to adjust the densities of the Kresling robots to be close to the density of water (1 g cm^{-3}) for better swimming performance. The magnetic plate in Supplementary Fig. 2a (2 mm thick) is designed for the single-plate robot, while the magnetic plate in Supplementary Fig. 8a (2.5 mm thick) with a larger volume is designed for the two-plate robot with better magnetic folding performance. The magnetic plates are fabricated by first mixing Ecoflex-0030 silicone rubber precursor (Smooth-On, Inc., USA), magnetic microparticles (NdFeB, average size 100 μm, Magnequench, Singapore), and glass bubbles (K20 series, 3M, USA) into a homogeneous mixture with a volume ratio of 7: 1: 2. The density of the mixture is calculated as 1.549 g cm^{-3}. The mixture is poured into 3D-printed PVA molds and cured at 80 °C for 0.5 h. Then, the PVA molds are dissolved in water. The fabricated magnetic plates are magnetized using a homemade magnetizer with a 1.5 T impulse magnetic field before assembling with Kresling patterns.

Kresling Robot Assembly. For all demonstrations except that of controllable medicine release, the Kresling robot is fabricated by assembling a Kresling pattern (Design 2, $H = 4.82$ mm) and a magnetic plate using Sil-poxy adhesive (Smooth-On, Inc., USA), as shown in Supplementary Fig. 2b. One fabricated sample is shown in Supplementary Fig. 2c. For the demonstration of controllable medicine release, the assembly components of the Kresling robot are shown in Supplementary Fig. 8b. The cavity-side of one magnetic plate is sealed with a Mylar hexagon while a 1.5 mm long needle is fixed to the middle of the opposite side of the plate. A 3 mm tall dye container is made by a heat shrink tube with an inner diameter of 2 mm. The container is filled with concentrated gel-based teal dye and the top end is sealed with Parafilm (Bemis Company, Inc., USA). All components are then assembled using Sil-poxy adhesive. During the folding process, the needle will puncture the parafilm and the "medicine" will be released. One fabricated sample is shown in Supplementary Fig. 8c.

2.Magnetic Actuation Setup

3D Helmholtz Coils. The locomotion of the Kresling robot is controlled under a three-dimensional (3D) magnetic field, which is generated by customized 3D Helmholtz coils shown in Supplementary Fig. 3. Three pairs of standard Helmholtz coils are configured orthogonally to each other. The magnetic field direction and strength can be manipulated by controlling the currents in three pairs of coils. The coils can generate 2.96 mT A^{-1}, 2.97 mT A^{-1}, and 2.90 mT A^{-1} uniform magnetic fields within a space of 160 mm by 120 mm by 80 mm (X-axis, Y-axis, and Z-axis, respectively).

Permanent Magnet for Magnetic Folding. A cylindrical N52 neodymium permanent magnet with a diameter of 50 mm and a thickness of 25 mm is used to fold the Kresling robot for controllable medicine release, as shown in Supplementary Fig. 9a. The relationship between the magnetic field strength and the distance from the center of the magnet is measured using a magnetometer (PCE-MFM 3000, PCE Instruments, USA) and shown in Supplementary Fig. 9b.

Uniformity of the Magnetic Field. The global XYZ coordinate is defined as shown in Supplementary Fig. 3. The XY-plane locates on the experimental substrate and is 30 mm from the center of the 3D coils. Finite element analysis (FEA, ANSYS Maxwell, ANSYS, Inc., USA) is performed to study the magnetic field distributions quantitatively. The magnetic field distributions (B_X, B_Y, and B_Z with 10 A in the coils) along the X-axis, Y-axis, Z-axis for multiple demonstrations are shown in Supplementary Fig. 15-17. B_X, B_Y, and B_Z are the strengths of magnetic fields generated by the X-axis, Y-axis, and

Z-axis coils. Here, we define the uniformity of the magnetic field in any axis as $B_{\min}/B_{\max}$, where $B_{\min}$ and $B_{\max}$ are the minimum and maximum fields within the range denoted by the grey regions in Supplementary Fig. 15-17.

For the demonstration of targeted delivery in Supplementary Fig. 15, the space occupied by the *ex vivo* pig stomach is 135 mm (X-axis, −67.5 mm to 67.5 mm) by 90 mm (Y-axis, −45 mm to 45 mm) by 20 mm (Z-axis, 0 mm to 20 mm). The uniformities of B_X, B_Y, and B_Z are 99.4%, 97.1%, and 91.6% along the X-axis, 99.9%, 99.6%, and 96.2% along the Y-axis, and 99.9%, 99.9%, and 99.4% along the Z-axis, respectively.

For the demonstration of swimming with a 3D spiral path in Supplementary Fig. 16, the space occupied by the swimming path is 110 mm (X-axis, −55 mm to 55 mm) by 120 mm (Y-axis, −60 mm to 60 mm) by 60 mm (Z-axis, 0 mm to 60 mm). The uniformities of B_X, B_Y, and B_Z are 99.9%, 98.6%, and 94.4% along the X-axis, 99.6%, 98.8%, and 93.3% along the Y-axis, and 99.9%, 99.9%, and 99.4% along the Z-axis, respectively.

For the demonstration of amphibious locomotion in the hybrid terrestrial-aquatic environment in Supplementary Fig. 17, the space occupied by the working environment is 145 mm (X-axis, −72.5 mm to 72.5 mm) by 85 mm (Y-axis, −42.5 mm to 42.5 mm) by 50 mm (Z-axis, 0 mm to 50 mm). The uniformities of B_X, B_Y, and B_Z are 99.2%, 96.2%, and 90.5% along the X-axis, 99.9%, 99.6%, and 96.5% along the Y-axis, and 99.9%, 99.9%, and 99.4% along the Z-axis, respectively.

3. Characterization of Materials and Kresling Samples

Mechanical Characterization of Polypropylene Film. Uniaxial tension tests are conducted on a universal testing machine (3344, Instron, Inc., USA) to characterize the mechanical property of polypropylene film used to fabricate the Kresling samples. A 100 N load cell is used. Thin-film samples (gauge zone 15 mm by 5 mm by 0.05 mm) are stretched to 5% strain at a strain rate of 0.01 s^{-1}, as shown in Supplementary Fig. 4. The Young's modulus is calculated to be 1577.9 MPa based on 0.5% strain, which can provide relatively large stiffness for Kresling's panels.

Mechanical Characterization of Kresling Samples. To measure the force needed to fold Kresling samples, we perform compression tests along the Kresling's longitudinal direction using a universal testing machine, as shown in Supplementary Fig. 5a. We first conduct cyclic loading tests for one Kresling sample (Design 2, $H = 4.82$ mm) at a strain rate of 0.01 s^{-1}. Here, contraction is defined as $\Delta H/H$, where H is the initial height of the Kresling and ΔH is the height change during compression. From the measured force-contraction curves in Supplementary Fig. 5b, we observe that the mechanical responses stabilize after 400 compression cycles. Thus, to soften Kresling's hinges and obtain a consistent mechanical behavior, we manually compress samples for 400 cycles before performing mechanical characterization and assembling them with other components for magnetic actuation.

For each Kresling design, four samples are compressed with 60% strain at a strain rate of 0.01 s^{-1}. Supplementary Fig. 5c shows the measured force-contraction curves of Kresling samples (Design 2, $H = 4.82$ mm) under compression along the longitudinal direction. By integrating the force-contraction curves, we obtained the stored energy during the compression process (Supplementary Fig. 5d). The Kresling shows coupled contraction and rotation behavior, which means that it can be folded under either a compressive force or a pair of torques. To derive the torque-rotation angle relationship from the force-contraction curve, the contraction-rotation angle (ψ) relation is needed, which is obtained by simulating the Kresling contraction using FEA (ABAQUS, Dassault Systèmes, France) and is shown in Supplementary Fig. 5e. By deriving the stored energy with respect to ψ, we can obtain the torque needed to fold the Kresling (Supplementary Fig. 5f). The measured force-contraction curves of four Kresling designs with different heights (Supplementary Table 1) are shown in Supplementary Fig. 18.

Magnetic Property Characterization of Magnetic Plates. The magnetic properties of the magnetic plates are measured using a vibrating sample magnetometer (7400A, Lake Shore Cryotronics, Inc., USA). The magnetic moments along the magnetization direction are measured using 4 mm × 4 mm × 1 mm magnetic plate samples with 10 vol% of magnetic particles. The corresponding remanent magnetization (M_r) is calculated by dividing the magnetic moment by the sample volume. The measured average M_r of magnetic plates is 58.21 kA m^{-1}.

4. Folding of Kresling Robot via Magnetic Actuation

Folding Measurement. To fold Kresling (Design2, $H = 4.82$ mm) via magnetic actuation, two magnetic plates (2.5 mm thick) are attached to generate a pair of magnetic torques, as shown in Supplementary Fig. 10. The magnetizations of two magnetic plates, denoted as $\mathbf{M}_1$ and $\mathbf{M}_2$, are in the plane of the magnetic plates with an initial relative angle of β (60°, 90°, and 120°). The applied magnetic field strength ranges from 0 to 270 mT by controlling the distance from the sample to the center of the magnet. Three samples are tested for each β, and the measured average contraction-magnetic field results are shown in Supplementary Fig. 10. For a specific angle β, the contraction increases with the magnetic field strength. Meanwhile, a larger β requires a smaller magnetic field to achieve the same amount of contraction.

Folding Calculation Based on Mechanical Characterization of Kresling Origami. For the Kresling with two magnetized plates, the force or torque needed to fold the structure will be influenced by the interaction force between magnetic plates. Therefore, longitudinal compression tests of the Kresling with magnetic plates are performed using a universal testing machine shown in Supplementary Fig. 11a. Here, β is set as 90° and four samples (Design2, $H = 4.82$ mm) are tested. The average

force-contraction curve and calculated torque-contraction curve are shown in Supplementary Fig. 11b, which will be used in the following calculation of magnetic folding.

When a magnetic field along the $\mathbf{M}_{\mathrm{net}}$ (vector sum of two plates' magnetizations) is applied, a pair of magnetic torques ($+T$ and $-T$) with same magnitude and opposite directions is generated, as shown in Supplementary Fig. 11c. In our experiments and calculations, the two magnetic plates have the same remanent magnetization M_{r} and volume V. The magnetic torque T can be then expressed as:

$$T\left(\Delta\beta_M\right) = BM_{\mathrm{r}}V\sin\Delta\beta_M = BM_{\mathrm{r}}V\sin\left(\beta_M - \beta_B\right), \qquad \text{[S1]}$$

where B is the strength of the magnetic field, $\Delta\beta_M$ is the angle between the magnetization of one magnetic plate and the magnetic field, β_M is the direction of the magnetization, and β_B is the direction of the magnetic field. The greatest achievable contraction of the Kresling under a specific magnetic field strength can be determined by the flowchart shown in Supplementary Fig. 11d. The structure can fold when the magnetic torque T is larger than the resistance torque (T_r, Supplementary Fig. 11b) needed. If the torque is not sufficient to overcome the resistance torque, current $\Delta\beta_M$ will be recorded and converted to the contraction based on the rotation angle-contraction relation in Supplementary Fig. 5e. The output contraction is what the sample can achieve under this specific magnetic field. The contractions under different magnetic field strengths can also be predicted using the same method. The steps for $\Delta\beta_M$ and B are chosen as 0.1° and 1 mT, respectively. The calculated folding results are compared with the experimental results, as shown in Supplementary Fig. 11e, demonstrating good agreements.

5. Experimental Details of Kresling Robot

Characterization of Jumping. The Kresling robot can jump when an instant magnetic field is applied in a different direction from the magnetization direction. The jumping height and distance are characterized with the same strength of 40 mT and different angles between magnetization and magnetic field (30°, 60°, 90°, 120°, and 150°). Three samples are tested for each angle. The measured average heights and distances are shown in Fig. 2f.

Swimming Speed Measurement of Kresling Robots with and without Hole and Cuts. The Kresling robots with and without hole and cuts are fabricated from different Kresling patterns (Design 2, $H = 4.82$ mm, Supplementary Fig. 1) to study the influence of the hole and cuts on robots' swimming performance. The swimming speeds of these two kinds of Kresling robots are measured with the magnetic field frequency ranging from 20 Hz to 30 Hz with 2 Hz interval and fixed 10 mT magnetic field strength. Three samples are tested for each kind of robot design. The measured average speeds are shown in Fig.3d.

Swimming Speed Measurement of Kresling Robots with Two Magnetic Plates. For the Kresling robots with two magnetic plates, the relative angle β between the magnetization directions of two magnetic plates influences the swimming performance. We fabricate the two-plate robots based on the Kresling with $H = 4.82$ mm and magnetic plates with a thickness of 2.5 mm. The swimming speeds of two-plate robots with different β (60°, 90°, and 120°) are measured with the magnetic field frequency ranging from 20 Hz to 30 Hz with 2 Hz interval and fixed 10 mT magnetic field strength. Three samples are tested for each β, and the measured average speeds are shown in Supplementary Fig. 12. With the increase of β, the swimming speed at the same frequency gradually decreases.

Demonstration of Targeted Medicine Delivery in Water. The maze shown in Supplementary Fig. 13 is used to illustrate the targeted medicine delivery in water. For the swimming locomotion, the strength and frequency of the applied magnetic field are 10 mT and 24 Hz. The magnetic field strength for medicine release is about 200 mT.

Demonstration of Amphibious Locomotion. In the demonstration of amphibious locomotion (Fig. 4), different magnetic profiles are used for different locomotion modes. For the self-adaptive locomotion (rolling or flipping), the strength and frequency of the applied magnetic field are 10 mT and 2 Hz. For the jumping, the magnetic field strength is 20 mT, and the angle between magnetization and the applied magnetic field is 120°. For the locomotion before submerging and after leaving the water, the strength and frequency of the applied magnetic field is 10 mT and 2 Hz. For the swimming locomotion (underwater or at air-water interfaces), the strength and frequency of the applied magnetic field is 10 mT and 24 Hz.

Swimming Speed Measurement of Kresling Robots with Different Heights. The geometry of the Kresling origami plays a critical role in the swimming performance of the Kresling robot. Here, we characterize the swimming speeds of Kresling robots with different geometries (different heights and tilted panels, Supplementary Table 1) to study the influence of Kresling geometry. To exclude the influences from cut, holes, and fabrication error, Kresling samples are 3D printed with Rigid Resin (Form 2, Formlabs Inc., USA) and attached with 2 mm thick magnetic plates, as shown in Supplementary Fig. 20a. In this way, the propulsion of the Kresling robot is only from the propeller-like structure. The swimming speeds are measured with the magnetic field frequency ranging from 20 Hz to 30 Hz with 2 Hz interval and fixed 10 mT magnetic field strength. Three samples are tested for each kind of robot design. The measured average speeds are shown in Supplementary Fig. 20b.

6. Selection of the Kresling Design for Demonstrations

In this paper, the selection of the Kresling design for demonstrations considers several different aspects, including structural symmetry, swimming performance, and folding performance. From the measurement results in Supplementary Fig. 20b, the Kresling robot based on Design 2 ($H = 4.82$ mm) shows a much faster swimming speed than other designs. In addition, the robot folding performances of four designs are calculated based on their mechanical characterizations (Supplementary Fig. 18). The calculated contraction-magnetic field curves are shown in Supplementary Fig. 19. It can be seen that the curves of Design 3 and Design 4 both have snapping points. In comparison, Kresling robots based on Design 1 and Design 2 show a smoother contraction process during the increase of the magnetic field strength, which is favorable for controllable medicine release. At the same time, Kresling robots based on Design 2 and Design 3 have an aspect ratio close to 1 (Supplementary Table 1), illustrating good global structural symmetry for on-ground locomotion. Based on the above analysis, Design 2 is selected for the demonstrations in the main text.

7. Angle Selection of the Kresling Robot with Two Magnetic Plates

The selection of the relative angle β of the Kresling robots with two magnetic plates is based on folding efficiency and swimming performance. In this paper, $\beta = 90°$ is chosen for both sufficient contraction and good swimming performance of the Kresling robot for targeted medicine delivery in water. Although magnetic plates with $\beta = 120°$ can enable a relatively large contraction (Supplementary Fig. 10), a larger β leads to a less stable swimming performance with lower swimming speed (Supplementary Fig. 12). On the contrary, the Kresling robot with $\beta = 60°$ shows good swimming performance, but it requires a very large magnetic field to achieve effective folding for medicine release. Therefore, $\beta = 90°$ is chosen to demonstrate controllable medicine release, with good swimming and folding performances.

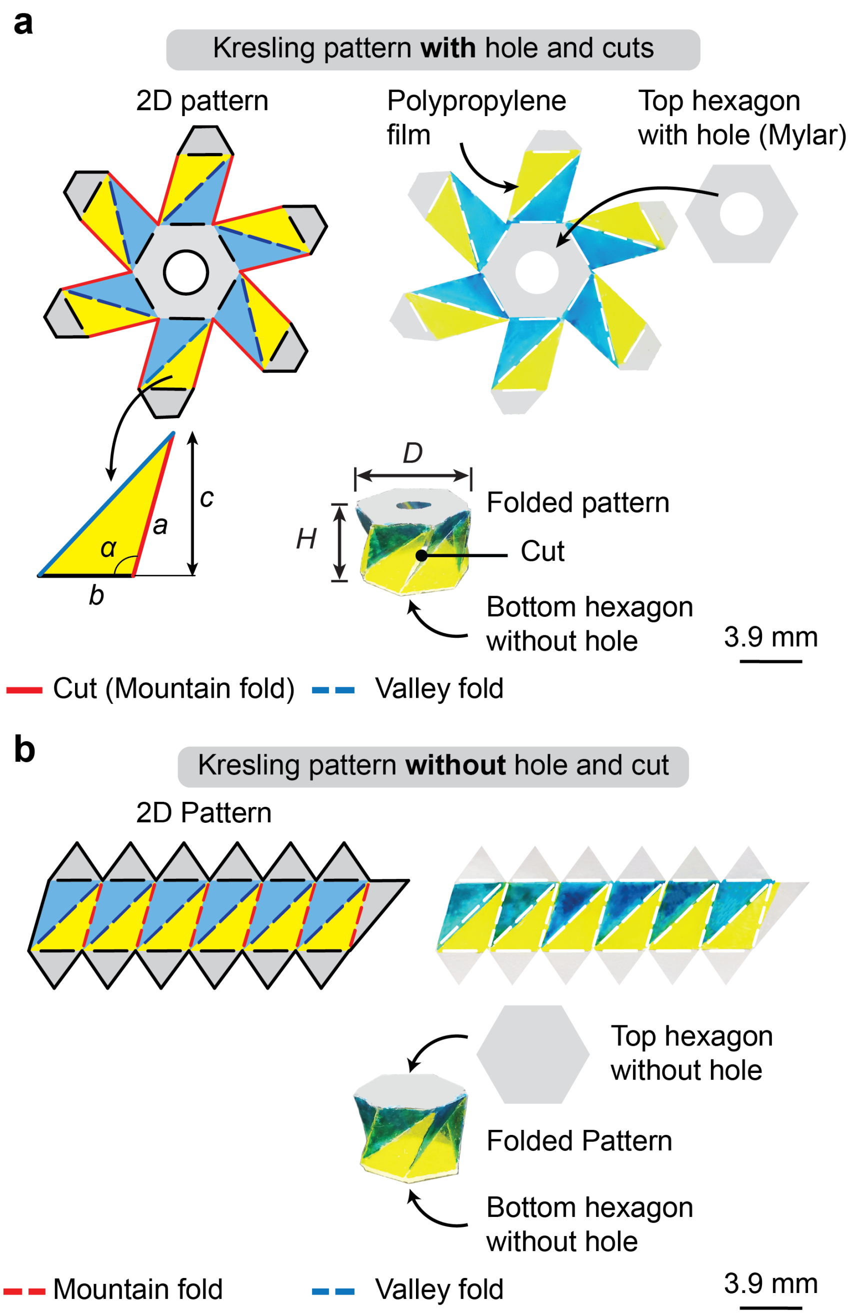

Fig. 1. Geometry, materials, patterns, and fabrication processes of Kresling origami. Kresling patterns (a) with and (b) without hole and cuts.

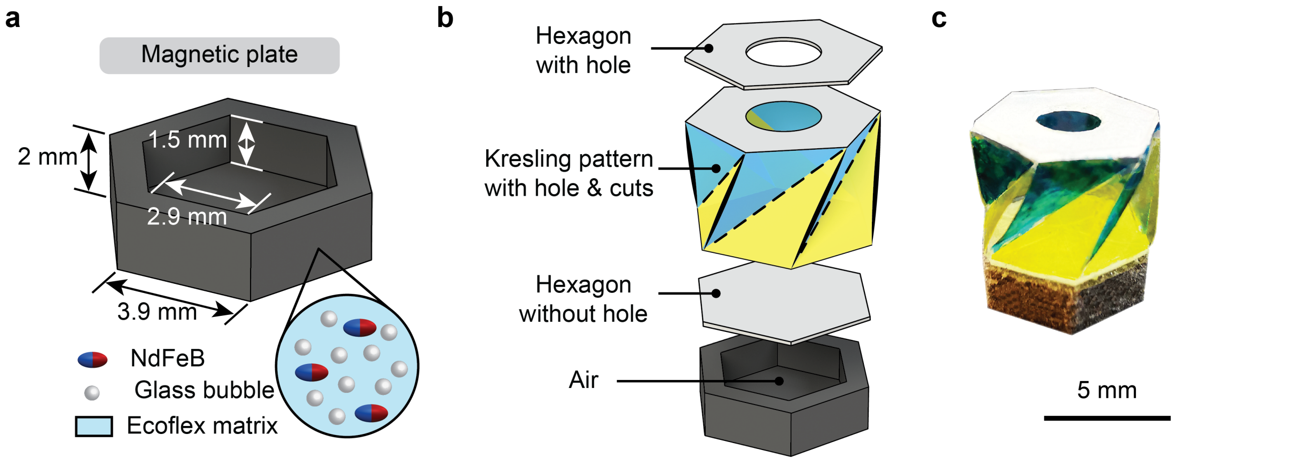

Fig. 2. Kresling robot by assembling Kresling pattern and magnetic plate. (a) Geometry and composition of the magnetic plate. (b) Exploded view of the Kresling robot. (c) Photo of the fabricated Kresling robot.

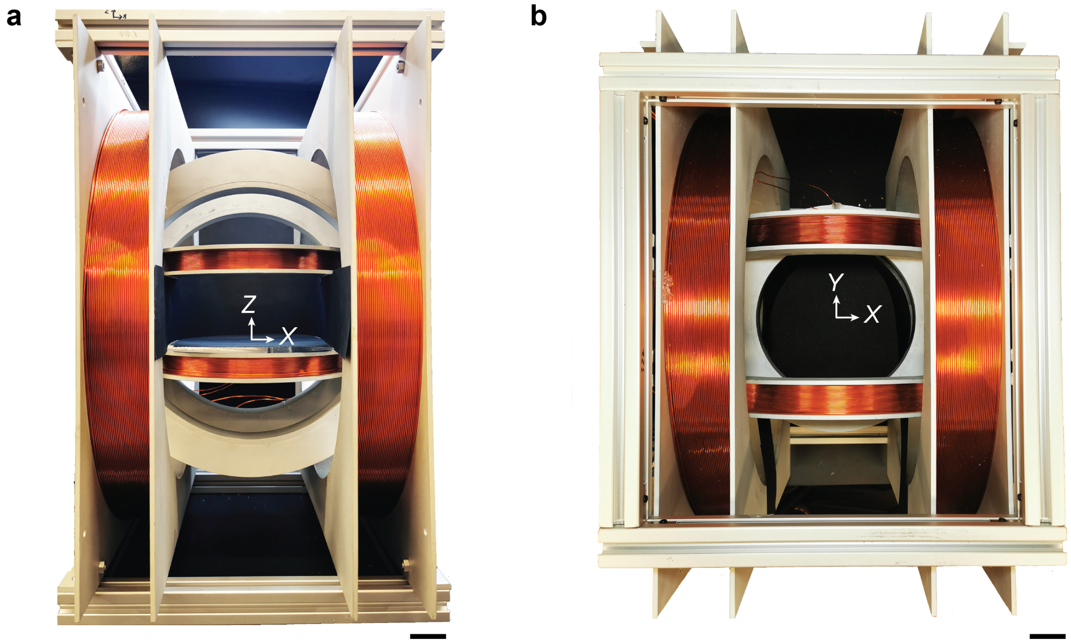

Fig. 3. 3D Helmholtz coils for magnetic actuation. (a) Front view. (b) Top view. Scale bars: 4 cm.

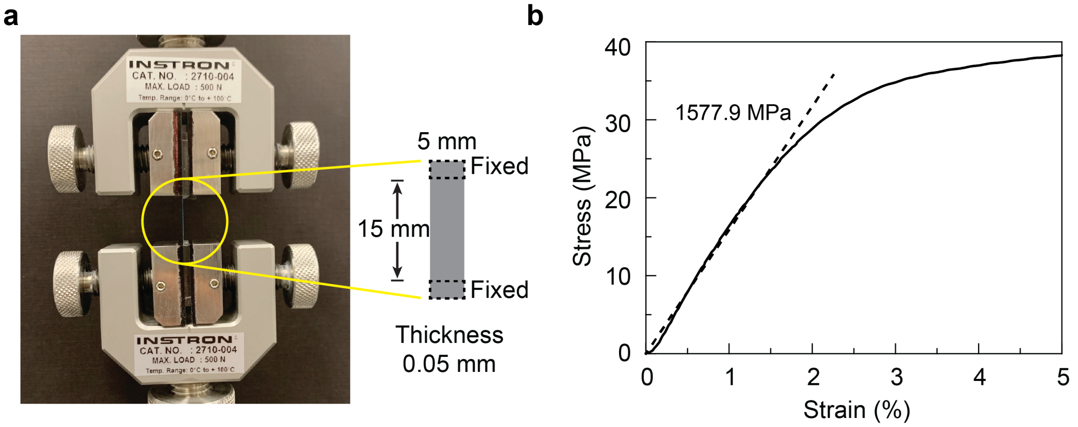

Fig. 4. Uniaxial tension tests of the polypropylene film used to fabricate Kresling samples. (a) Experimental setup and the dimension of the tested polypropylene film. (b) Tension test results of polypropylene film with 5% strain at a strain rate of 0.01 s^{-1}. The Young's modulus is calculated to be 1577.9 MPa based on 0.5% strain.

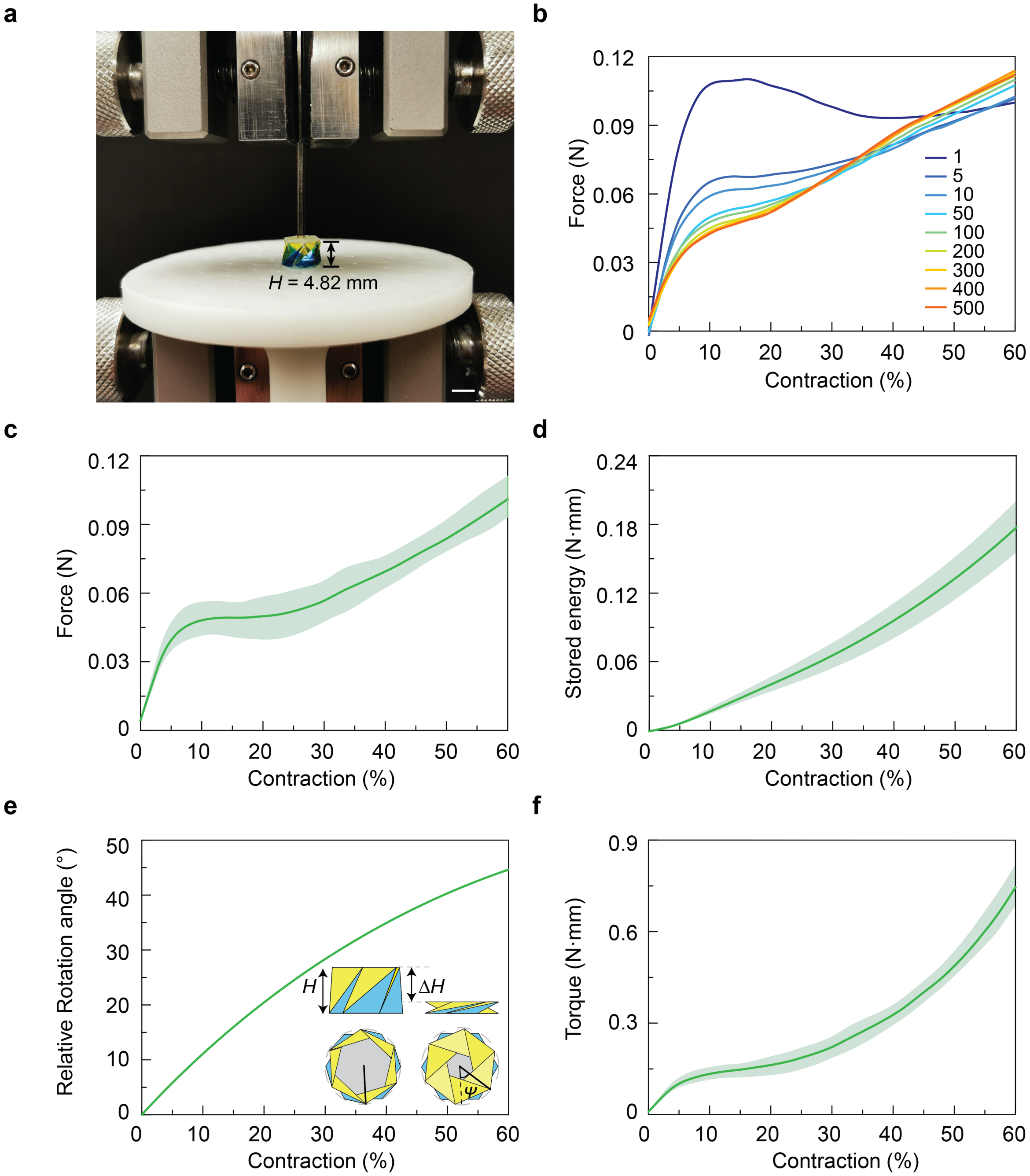

Fig. 5. Mechanical characterization of Kresling origami under longitudinal compression. (a) Experimental setup for Kresling compression. Scale bar: 5 mm. (b) Experimental force-contraction ($\Delta H/H$) curve of one Kresling sample (H = 4.82 mm) up to 500 contraction cycles. (c) Experimental force-contraction curve of Kresling samples after 400 cycles' compression along the longitudinal direction. (d) Stored energy-contraction curve by integrating measured force-contraction results. (e) Relationship between relative rotation angle ψ and contraction obtained via FEA. (f) Calculated torque-contraction curve. Solid lines are the averaged responses of four Kresling samples, and shaded regions represent the range of responses.

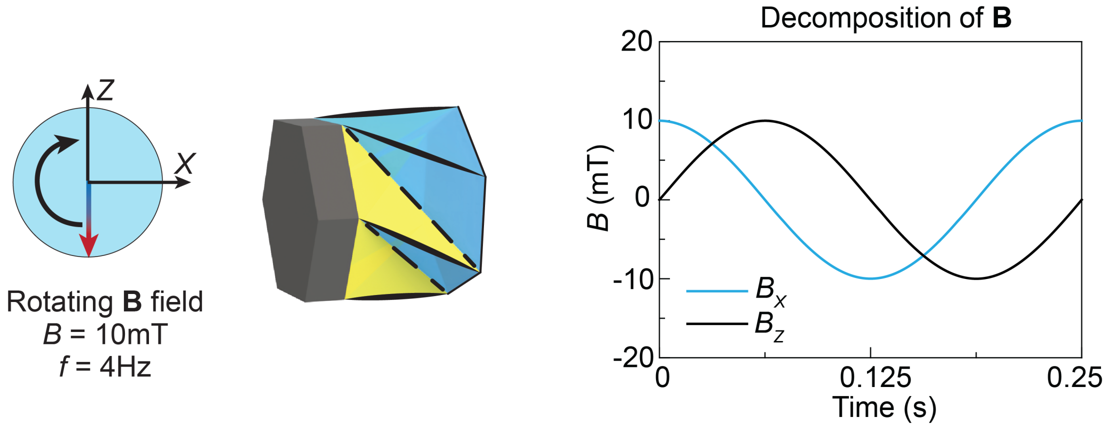

Fig. 6. Rotating magnetic field profile with a strength of 10 mT and frequency of 4 Hz.

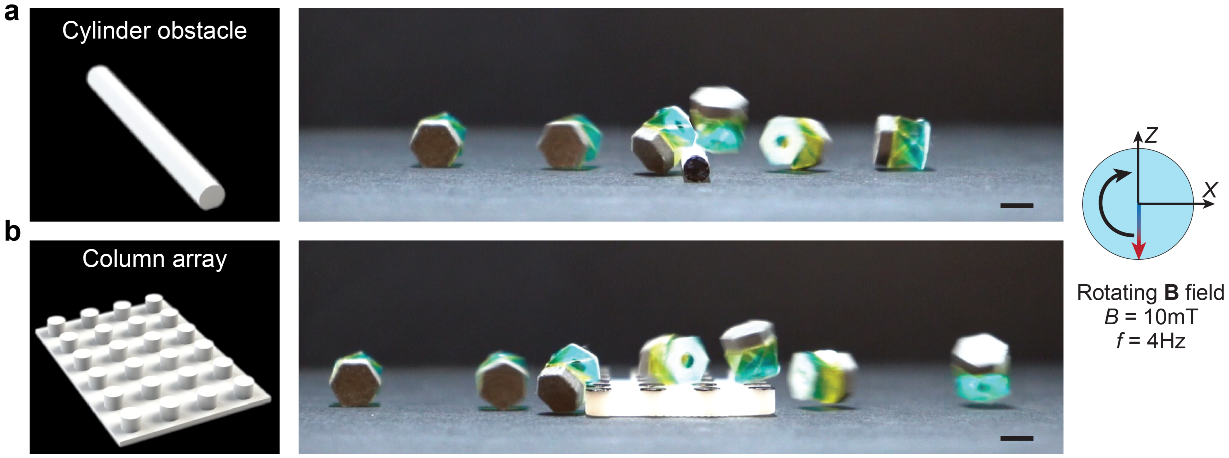

Fig. 7. Self-adaptive locomotion on different terrains. Demonstration of self-adaptive locomotion on the terrains with (a) a cylinder obstacle and (b) a column array. Scale bars: 5 mm.

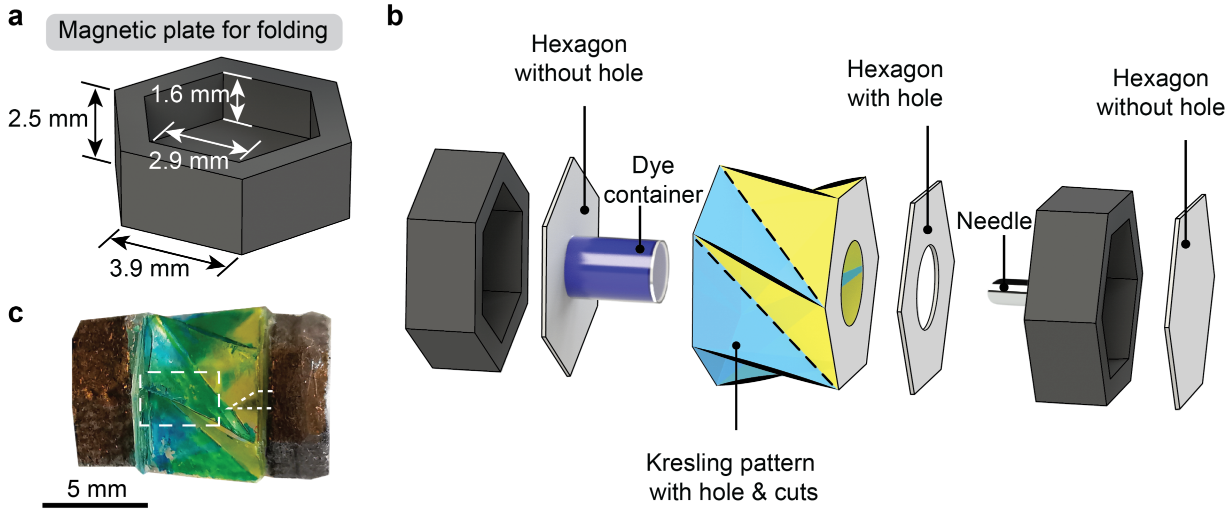

Fig. 8. Fabrication process of the Kresling robot for controllable medicine release. (a) Geometry of the magnetic plate for folding. (b) Exploded view of the Kresling robot. (c) Photo of the fabricated Kresling robot.

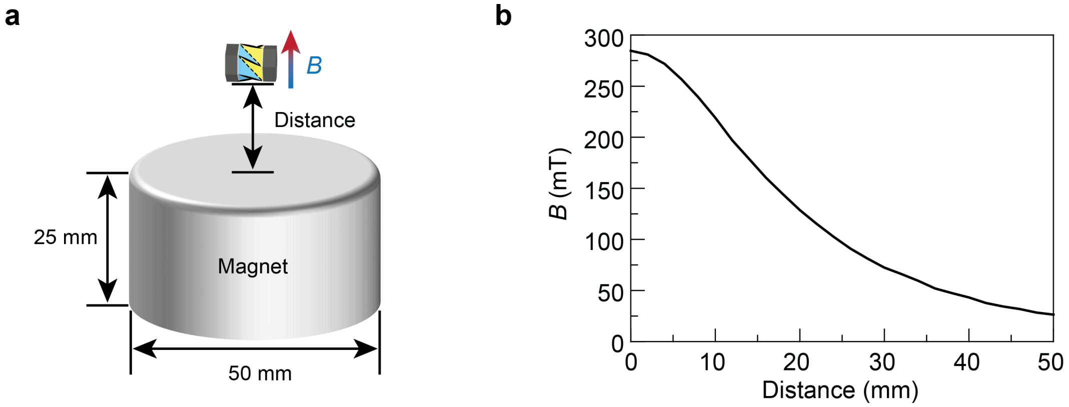

Fig. 9. Characterization of the magnetic field of the permanent magnet for folding of the robot. (a) Schematic of magnetic folding actuated by a N52 magnet. (b) The relationship between the magnetic field strength and the distance from the center of the magnet.

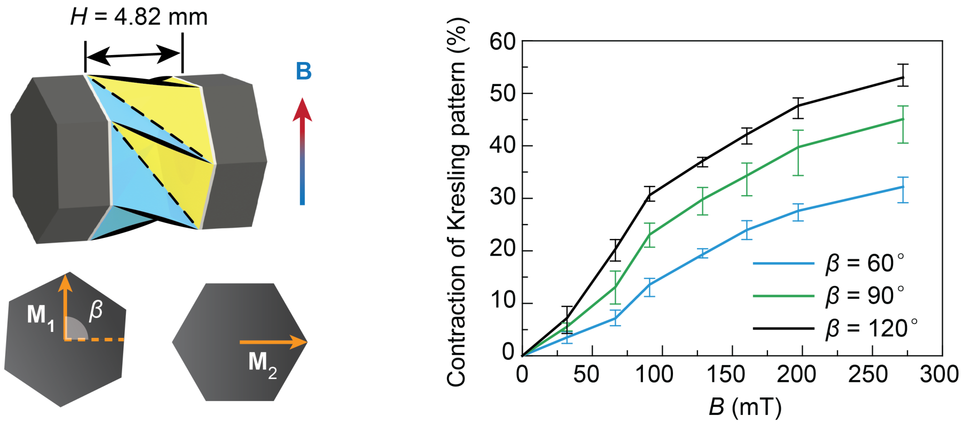

Fig. 10. Folding of Kresling robots. β indicates the angle between the magnetization directions of two magnetic plates.

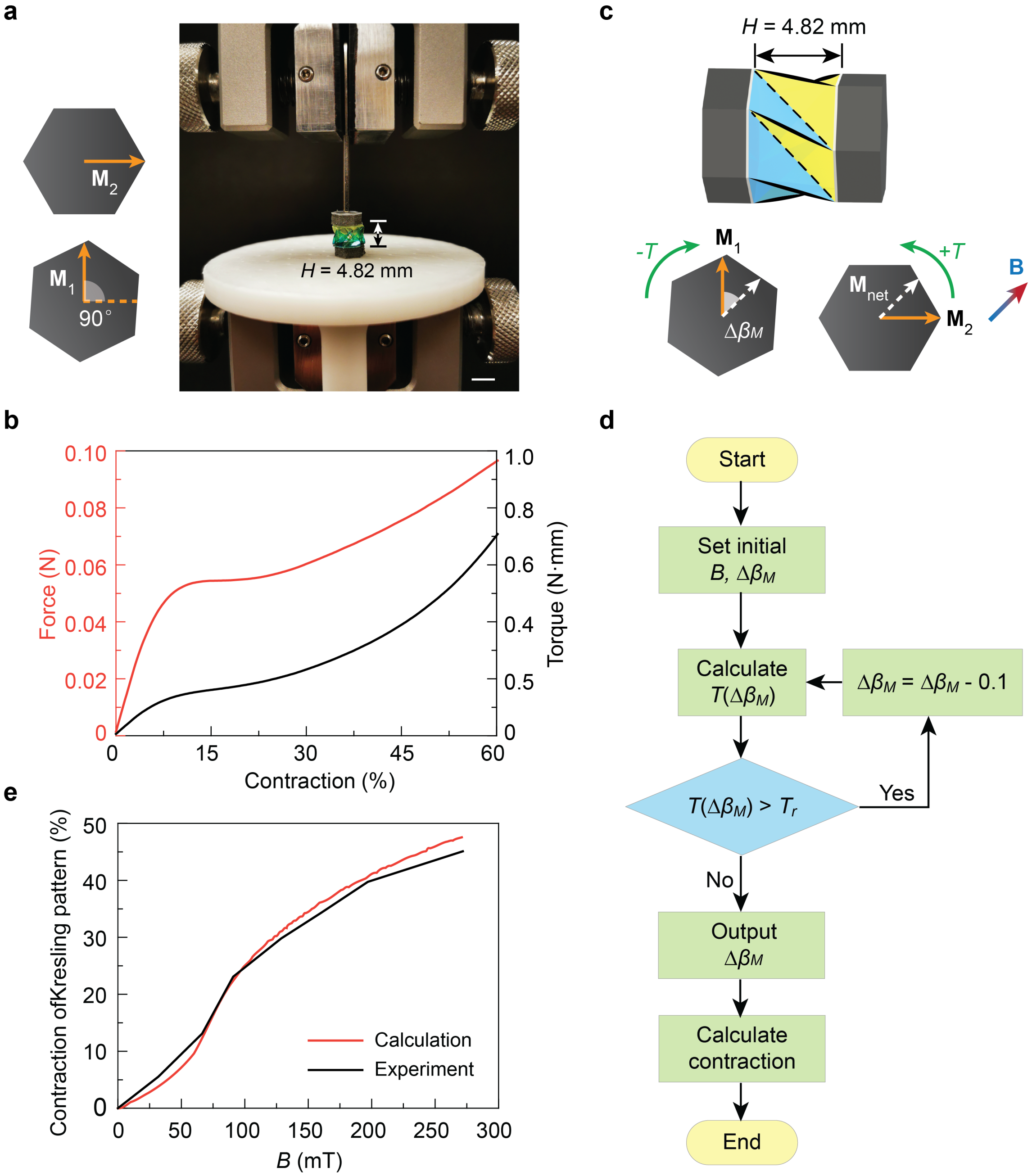

Fig. 11. Calculation of the robot folding under magnetic actuation. (a) Compression test of the Kresling robot with two magnetic plates. (b) Measured force-contraction curve of the sample (H = 4.82 mm) with a β of 90° and converted torque-contraction curve. The calculated torque is defined as the resistance torque (T_r) for folding the robot. (c) Schematic of the robot folding. $\Delta\beta_M$ indicates the angle between the magnetization direction of one magnetic plate and the applied magnetic field direction during folding process. (d) Flowchart of the calculation algorithm for achieving the contraction under a specific magnetic field strength. The initial $\Delta\beta_M$ is 45° when β = 90°. (e) Comparison of experimental and calculation results of the robot folding.

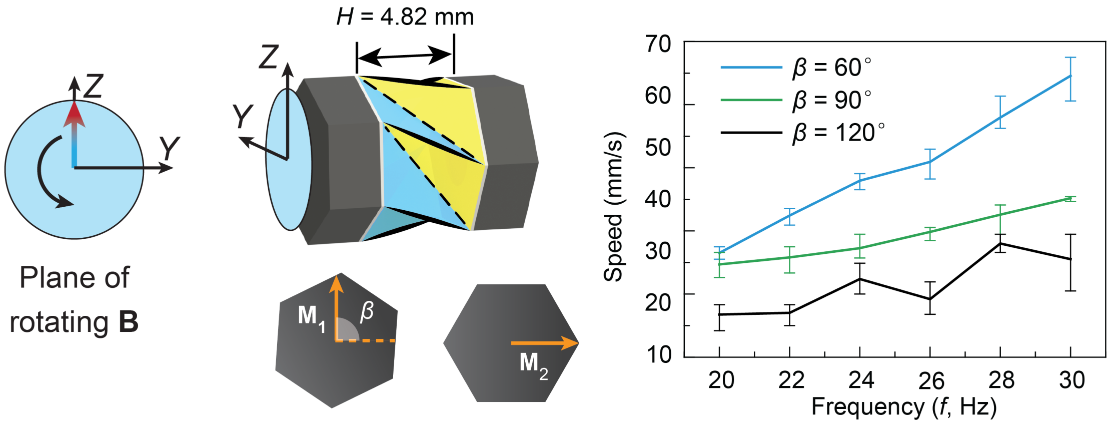

Fig. 12. Swimming performance of the Kresling robot with two magnetic plates. β indicates the angle between the magnetization directions of two magnetic plates.

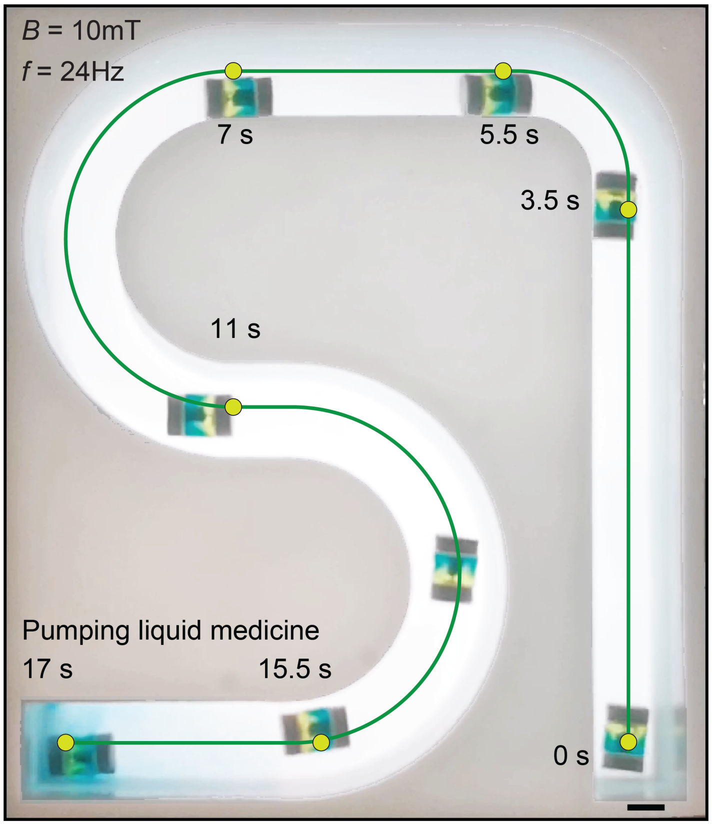

Fig. 13. Demonstration of targeted delivery of liquid medicine in water by the Kresling robot (Supplementary Movie 7). Scale bar: 5 mm.

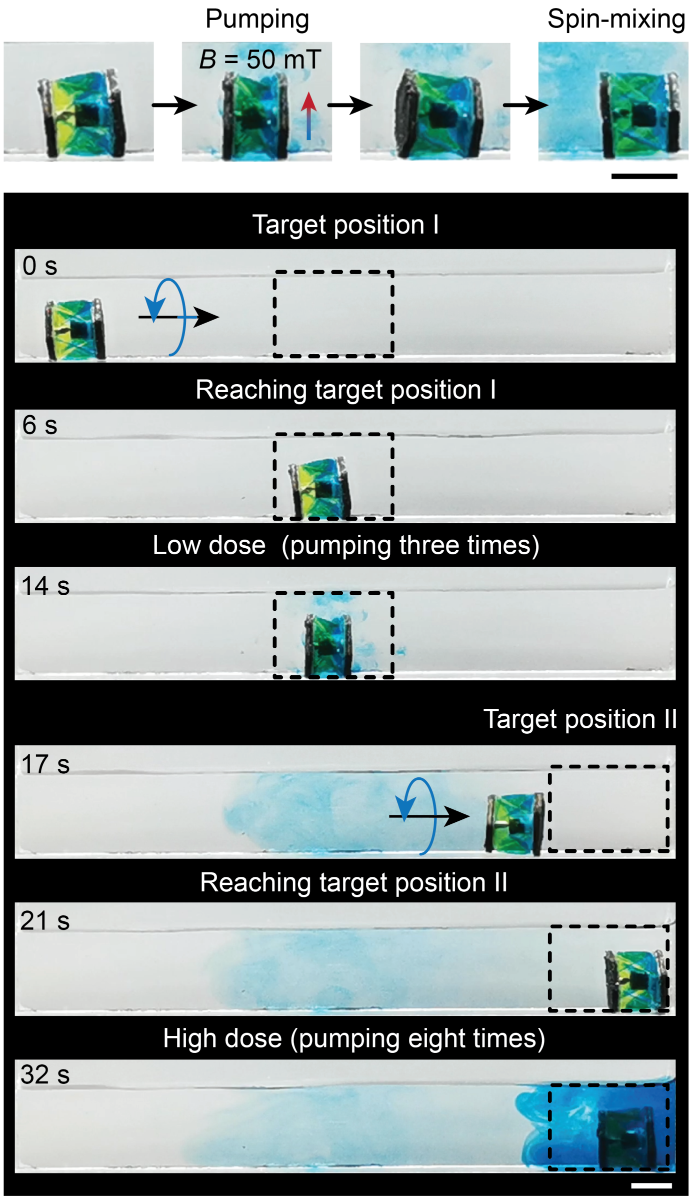

Fig. 14. Controlled delivery of liquid medicine at two target positions by a large-scale Kresling robot (Supplementary Movie 8). Scale bars: 8 mm.

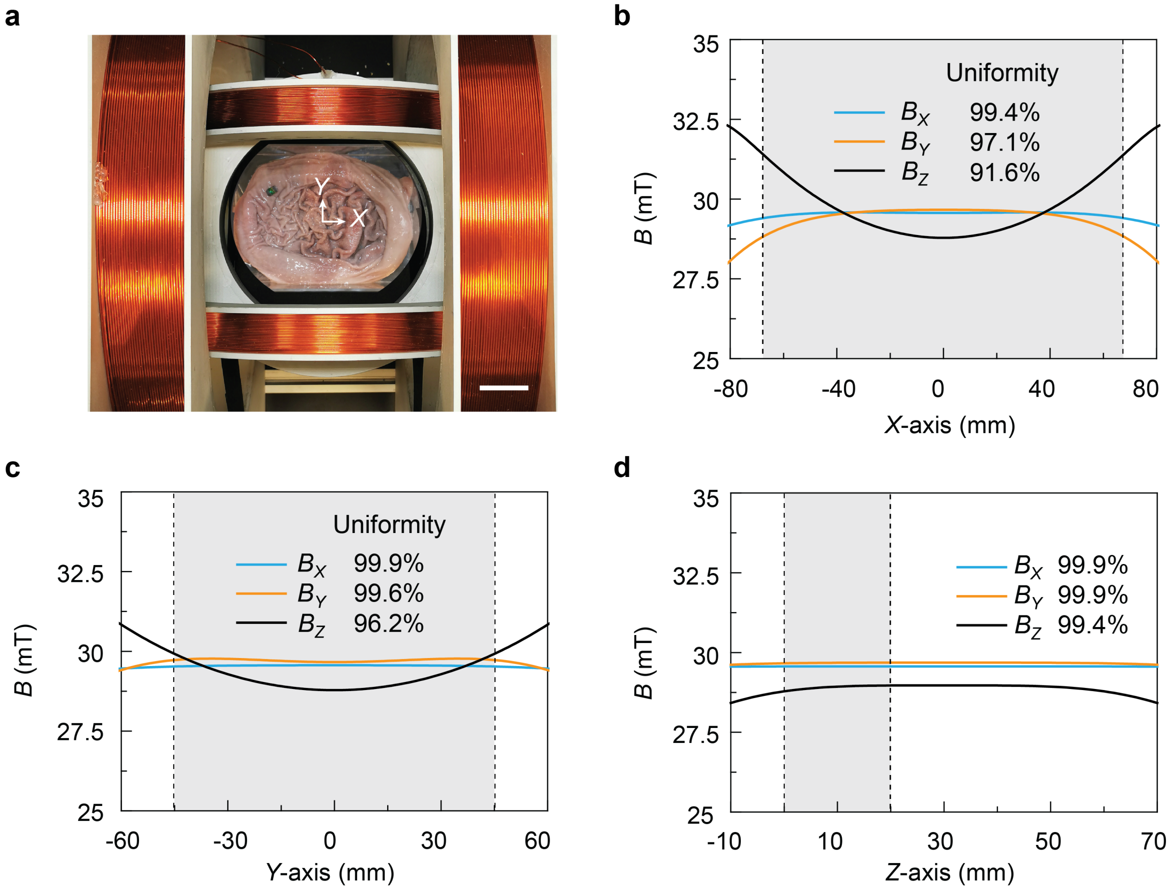

Fig. 15. The magnetic field uniformity for the demonstration of targeted delivery of liquid medicine in an *ex vivo* pig stomach. (a) Experimental setup. Scale bar: 4 cm. The magnetic field uniformity along (b) X-axis, (c) Y-axis, and (d) Z-axis. The grey regions indicate the maximum working range for the demonstration.

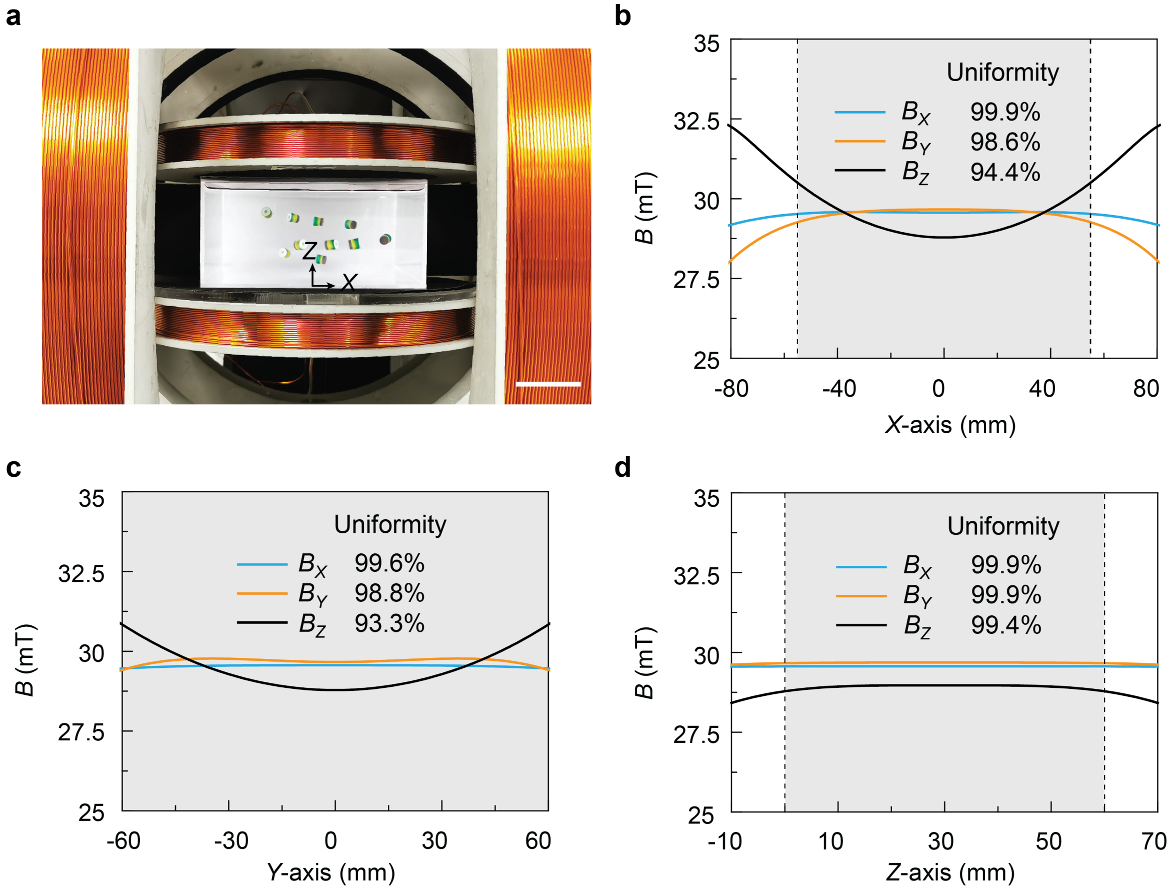

Fig. 16. The magnetic field uniformity for the demonstration of swimming with a 3D spiral path. (a) Experimental setup. Scale bar: 4 cm. The magnetic field uniformity along (b) *X*-axis, (c) *Y*-axis, and (d) *Z*-axis. The grey regions indicate the maximum working range for the demonstration.

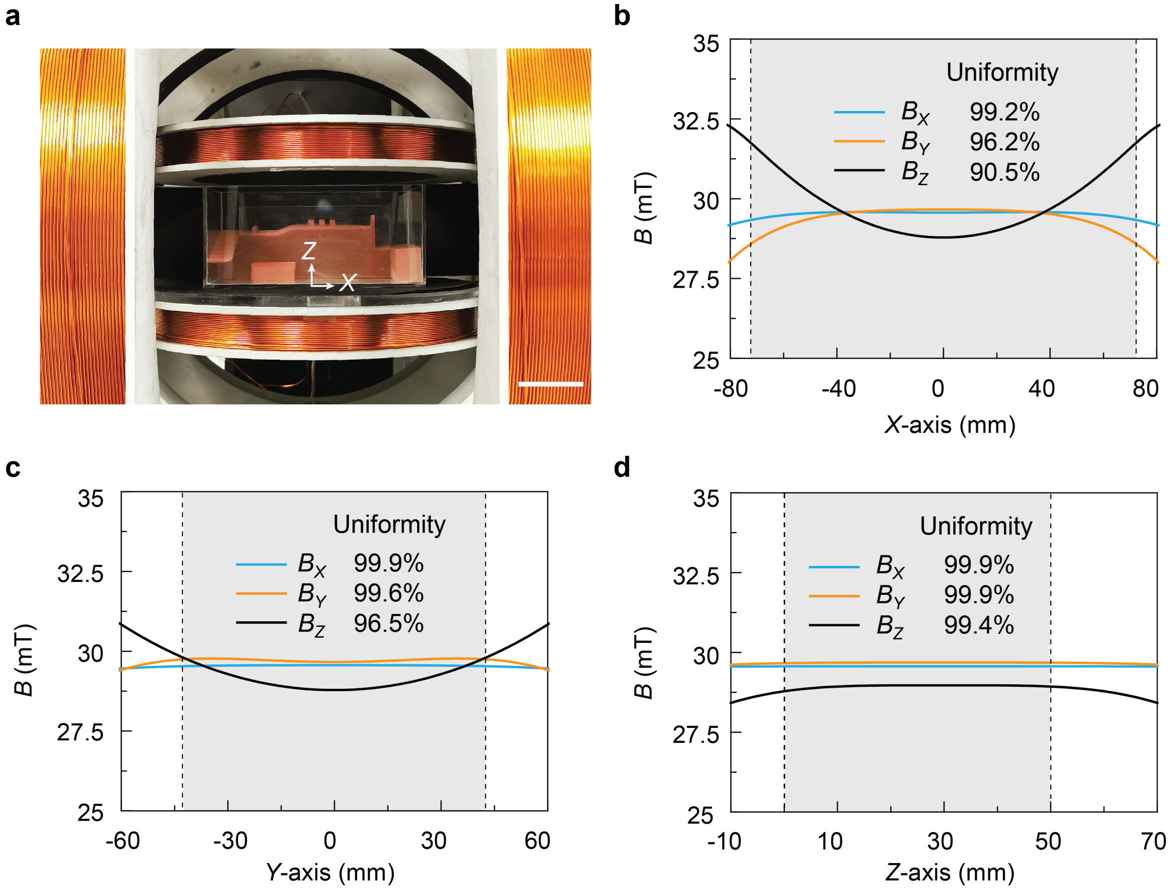

Fig. 17. The magnetic field uniformity for the demonstration of amphibious locomotion in the hybrid terrestrial-aquatic environment. (a) Experimental setup. Scale bar: 4 cm. The magnetic field uniformity along (b) *X*-axis, (c) *Y*-axis, and (d) *Z*-axis. The grey regions indicate the maximum working range for the demonstration.

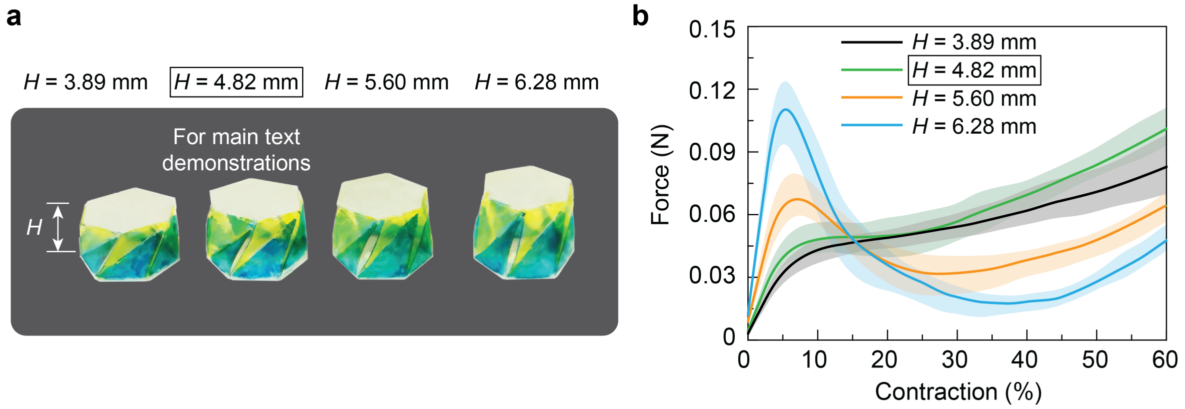

Fig. 18. Mechanical characterization of the four Kresling patterns with different heights. (a) Photos of four Kresling patterns with different heights. (b) Experimental force-contraction curves of four Kresling designs after 400 cycles' compression from the longitudinal direction. Solid lines are the averaged responses of four Kresling samples, and shaded regions represent the range of responses.

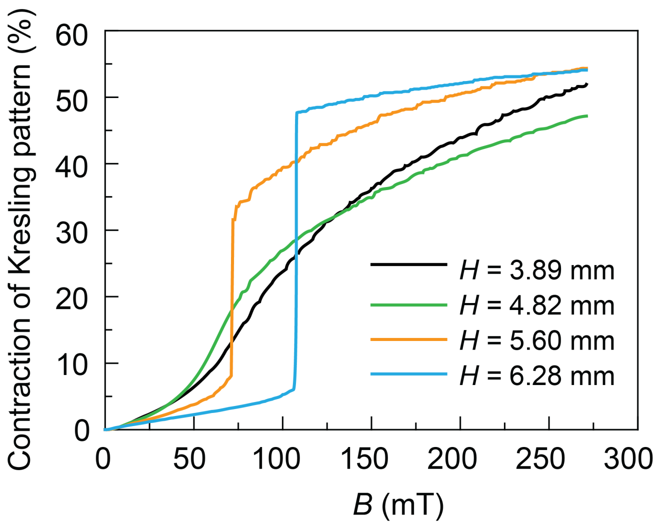

Fig. 19. Calculation results of robot folding for four Kresling designs with different heights based on their mechanical characterizations when β = 90°.

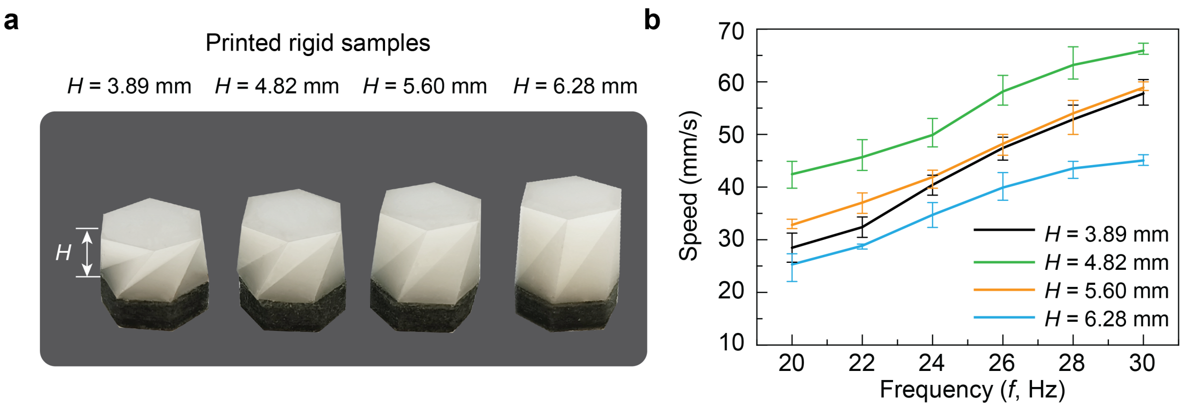

Fig. 20. Swimming performance of the Kresling robots with different heights. (a) Photos of four robots with printed Kresling samples. (b) Swimming speed comparison.

Table 1. Geometries of Four Kresling Origami Designs

Design	b (mm)	a (mm)	c (mm)	α (°)	H (mm)	Aspect ratio w/o magnetic plate	Aspect ratio w/ magnetic plate[1]
1	3.9	4.84	4.5	111.64	3.89	0.544	0.801
2 (used in the main text)	3.9	5.33	5.1	106.92	4.82	0.663	0.920
3	3.9	5.82	5.7	101.74	5.62	0.763	1.020
4	3.9	6.33	6.3	95.77	6.28	0.851	1.107

[1]The aspect ratio of the Kresling robot with one magnetic plate (2 mm thick).

Supplementary Movie 1. Self-adaptive on-ground locomotion

Supplementary Movie 2. Self-adaptive locomotion on different terrains

Supplementary Movie 3. Jumping mechanism

Supplementary Movie 4. Pumping mechanism for controlled delivery of liquid medicine in the pig stomach

Supplementary Movie 5. Underwater swimming mechanism

Supplementary Movie 6. Underwater swimming with 2D and 3D paths

Supplementary Movie 7. Targeted delivery of liquid medicine in water

Supplementary Movie 8. Targeted delivery of liquid medicine in water by a large-scale robot

Supplementary Movie 9. Swimming at air-water interfaces

Supplementary Movie 10. Sucking mechanism for capturing cargo

Supplementary Movie 11. Amphibious locomotion with cargo transportation

Supplementary Movie 12. Amphibious locomotion in the pig stomach containing viscous fluid

References

1. HC Yang, J Hou, V Chen, ZK Xu, Janus membranes: exploring duality for advanced separation. *Angewandte Chemie Int. Ed.* **55**, 13398–13407 (2016).
