# Supplementary material for: Spinning-enabled wireless amphibious origami millirobot
Source: Nat Commun. 2022 Jun 14;13:3118. doi: 10.1038/s41467-022-30802-w (PMC9198078; doi:10.1038/s41467-022-30802-w)
Supplement: Supplementary file 3 — Description of Additional Supplementary Files [file 41467_2022_30802_MOESM3_ESM.pdf]

Description of Additional Supplementary Files

File Name: Supplementary Movie 1
Description: Self-adaptive onground locomotion

File Name: Supplementary Movie 2
Description: Self-adaptive locomotion on different terrains

File Name: Supplementary Movie 3
Description: Jumping mechanism

File Name: Supplementary Movie 4
Description: Pumping mechanism for controlled delivery of liquid medicine in the pig stomach

File Name: Supplementary Movie 5
Description: . Underwater swimming mechanism

File Name: Supplementary Movie 6
Description: Underwater swimming with 2D and 3D paths

File Name: Supplementary Movie 7
Description: Targeted delivery of liquid medicine in water

File Name: Supplementary Movie 8
Description: Targeted delivery of liquid medicine in water by a large-scale robot

File Name: Supplementary Movie 9
Description: Swimming at airwater interfaces

File Name: Supplementary Movie 10
Description: Sucking mechanism for capturing cargo

File Name: Supplementary Movie 11
Description: Amphibious locomotion with cargo transportation

File Name: Supplementary Movie 12
Description: Amphibious locomotion in the pig stomach containing viscous fluid
